# Fanconi Anemia Pathway in Colorectal Cancer: A Novel Opportunity for Diagnosis, Prognosis and Therapy

**DOI:** 10.3390/jpm12030396

**Published:** 2022-03-04

**Authors:** Fatemeh Ghorbani Parsa, Stefania Nobili, Mina Karimpour, Hamid Asadzadeh Aghdaei, Ehsan Nazemalhosseini-Mojarad, Enrico Mini

**Affiliations:** 1 Basic and Molecular Epidemiology of Gastrointestinal Disorders Research Center, Research Institute for Gastroenterology and Liver Diseases, Shahid Beheshti University of Medical Sciences, Tehran 19857-17413, Iran; ghorbaniparsa@tabrizu.ac.ir (F.G.P.); hamid.asadzadeh@sbmu.ac.ir (H.A.A.); 2 Department of Neurosciences, Imaging and Clinical Sciences, University “G. D’Annunzio” Chieti-Pescara, 66100 Chieti, Italy; stefania.nobili@unich.it; 3 Center for Advanced Studies and Technology (CAST), University “G. D’Annunzio” Chieti-Pescara, 66100 Chieti, Italy; 4 Department of Genetics, Faculty of Biological Sciences, Tarbiat Modares University, Tehran 14115-154, Iran; mina.karimpour@modares.ac.ir; 5 Gastroenterology and Liver Diseases Research Center, Research Institute for Gastroenterology and Liver Diseases, Shahid Beheshti University of Medical Sciences, Tehran 19857-17413, Iran; 6 Department of Health Sciences, University of Florence, 50139 Florence, Italy; 7 DENOTHE Excellence Center, University of Florence, 50139 Florence, Italy

**Keywords:** colorectal cancer, Fanconi anemia, prognostic biomarker, predictive biomarker, target therapy, synthetic lethality

## Abstract

Colorectal cancer (CRC) is the third most commonly diagnosed malignancy and has the second highest mortality rate globally. Thanks to the advent of next-generation sequencing technologies, several novel candidate genes have been proposed for CRC susceptibility. Germline biallelic mutations in one or more of the 22 currently recognized Fanconi anemia (FA) genes have been associated with Fanconi anemia disease, while germline monoallelic mutations, somatic mutations, or the promoter hypermethylation of some *FANC* genes increases the risk of cancer development, including CRC. The FA pathway is a substantial part of the DNA damage response system that participates in the repair of DNA inter-strand crosslinks through homologous recombination (HR) and protects genome stability via replication fork stabilization, respectively. Recent studies revealed associations between FA gene/protein tumor expression levels (i.e., *FANC* genes) and CRC progression and drug resistance. Moreover, the FA pathway represents a potential target in the CRC treatment. In fact, *FANC* gene characteristics may contribute to chemosensitize tumor cells to DNA crosslinking agents such as oxaliplatin and cisplatin besides exploiting the synthetic lethal approach for selective targeting of tumor cells. Hence, this review summarizes the current knowledge on the function of the FA pathway in DNA repair and genomic integrity with a focus on the *FANC* genes as potential predisposition factors to CRC. We then introduce recent literature that highlights the importance of *FANC* genes in CRC as promising prognostic and predictive biomarkers for disease management and treatment. Finally, we represent a brief overview of the current knowledge around the *FANC* genes as synthetic lethal therapeutic targets for precision cancer medicine.

## 1. Introduction

Colorectal cancer (CRC) is the second most lethal cancer globally [1]. Approximately 20% to 30% of CRCs are potentially linked to genetic factors, although the most underlying genetic causes remain unexplained [2]. However, the Mendelian CRC syndromes with defined genetic predispositions account for approximately 5% to 10% of all CRC syndromes [3]. These well-defined hereditary CRCs are caused by pathogenic mutations or epimutations in DNA mismatch repair (MMR) genes—*MLH1*, *MSH2*, *MSH6*, and *PMS2* for nonpolyposis CRC cases and pathogenic variants (PVs) in *MUTYH* and *NTHL* (recessive inheritance) along with those in APC for adenomatous polyposis syndromes [2,4].

Other—less frequent—CRC-predisposing syndromes, characterized by the presence of hamartomatous polyps, are caused by mutations in *SMAD4*, *BMPR1A*, *STK11*, and *PTEN* [5]. Additionally, some syndromes are still being further characterized, such as the I1307K polymorphism in APC as well as the polymerase proofreading-associated polyposis (PPAP) caused by the germline mutations in *POLE* and *POLD1* [6,7,8,9]. There are also some other CRC syndromes, such as serrated polyposis syndrome, the causative genes of which are not fully understood [10]. However, besides these well-defined predisposing genes, several non-CRC hereditary cancer genes have been recently found to be mutated in CRC patients, which might be connected with an increased risk of CRC or adenomatous polyposis [5,11].

Interestingly, the knowledge of the microsatellite status (i.e., MSS or MSI), strictly related to an impaired MMR system, is today widely informative not only in terms of CRC predisposition (i.e., Lynch syndrome diagnosis) but also in terms of prognosis and therapeutic options. In fact, MSI identifies a subset of CRC patients (i.e., low-risk stage II patients) at better prognosis who do not achieve advantages from adjuvant chemotherapy. In addition, the MSI-H condition is a prominent biomarker for the treatment of several tumors, including metastatic CRC, with immune checkpoint inhibitors. Thus, the evaluation of MMR/MSI has become part of the standard diagnostic and therapeutic course in CRC. Therefore, the major oncological international societies recommend the use of immune checkpoint inhibitors in the treatment of metastatic CRC either as first-line treatment or as subsequent therapy after the front-line treatment [3,12,13].

Fanconi anemia (FA) is a rare autosomal recessive genetic disease characterized by bone marrow failure, cancer susceptibility, and developmental abnormalities that originate from biallelic mutations in at least one of 22 *FANC* genes (designated as FA complementation groups), which have been identified so far [14]. The 22 FANC genes are *FANCA*, *FANCB*, *FANCC*, *FANCD1/BRCA2*, *FANCD2*, *FANCE*, *FANCF*, *FANCG*, *FANCI*, *FANCJ/BRIP1*, *FANCL*, *FANCM*, *FANCN/PALB2*, *FANCO/RAD51C*, *FANCP/SLX4*, *FANCQ/ERCC4*, *FANCR/Rad51*, *FANCS/BRCA1*, *FANCT/UBE2T*, *FANCU/XRCC2*, *FANCV/REV7* and *FANCW/RFWD3* [15]. All the FA genes show autosomal recessive inheritance except for the X-linked *FANCB* gene and *FANCR/RAD51* gene, whose dominant mutations are associated with FA-like syndrome [16,17].

The products of these 22 *FANC* genes cooperate in a cellular repair pathway known as the FA pathway or the FA-BRCA pathway, emphasizing that some of the FA proteins are BRCA-related proteins [18]. This pathway plays a pivotal role in the repair of DNA inter-strand crosslink by the combined actions of nucleotide excision repair (NER), homologous recombination (HR), and trivial involvement of the translesion DNA synthesis (TLS) pathway [19,20]. DNA inter-strand crosslinks are covalent linkages between two complementary strands of DNA that block DNA strand separation upon replication and transcription. Unresolved DNA inter-strand crosslink creates clastogenic effects leading to genomic instability, a critical event in the accumulation of genetic mutations, which trigger cancer initiation [21,22].

The connection between the FA pathway and malignancy was evidenced when mutations in *FANCD1/BRCA2*, the breast/ovarian cancer susceptibility gene, had been detected in FA patients [20,23]. It is known that the biallelic mutations in *FANC* genes lead to Fanconi anemia disease, while germline monoallelic mutations, somatic mutations, or promoter hypermethylation of some *FANC* genes increase the risk of cancer in non-FA individuals [24,25]. For instance, it is well established that monoallelic mutations in *FANCS/BRCA1* and/or *FANCD1/BRCA2* are associated with breast and ovarian cancer susceptibility [17]. Genomic alterations that involve other genes in HR pathways, including *FANCJ/BRIP1* and *FANCN/PALB2*, have also been suggested to increase the lifetime risk of epithelial ovarian cancer development [26]. Recently, the identification of mutations in *FANC* genes such as *FANCD1/BRCA2* [27,28], *FANCJ/BRIP1* [27,29], *FANCN/PALB2* [27,30], and *FANCA* [29,31], among CRC patients who did not harbor detectable mutations in known CRC susceptibility genes, highlights the role of *FANC* genes as potential CRC predisposition genes.

Thus, this narrative review aims to provide an overview of the function of *FANC* genes in CRC predisposition, progression, and drug resistance, in addition to their potential clinical value as predictive and/or prognostic biomarkers, after representing a summary of the FA pathway role in the repair of DNA lesions and the protection of genomic stability. However, investigation into the function of *FANC* genes in CRC occurrence is still in its infancy. To our knowledge, this review is the first attempt to summarize current knowledge on the implication of *FANC* genes in CRC. Although there is little evidence regarding this subject currently, we assume that more information will emerge in the next future.

## 2. The Fanconi Anemia Pathway in DNA Repair and Maintenance of Genome Integrity

The FA pathway is a biochemical network that actively participates in the DNA repair and genome integrity maintenance processes through resolving DNA inter-strand crosslinking damages, participating in replication fork stability, and cytokinesis [32]. Of these, its canonical function is the inter-strand crosslink repair [33].

### 2.1. The FA Pathway and Inter-Strand Crosslink Repair

**Figure 1:**
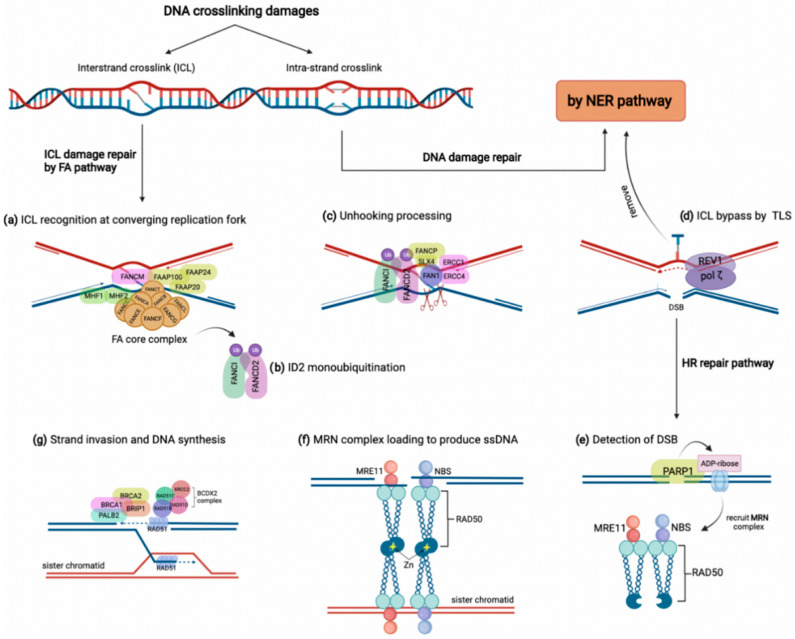
The FA pathway and inter-strand crosslink (ICL) repair. DNA crosslinking damages generally include intra-strand and inter-strand crosslink damages, which are repaired by NER and FA pathways, respectively. (**a**) In the FA pathway, the ICL damage is recognized by FANCM accompanying some other FAAPs at converging replication fork, which results in FA core complex loading along with FAAP100, FAAP20, and FAAP24, as well as MHF1 and MHF2. (**b**) FA core complex activates the ID2 complex by monoubiquitylation of FANCI and FANCD2. (**c**) Monoubiquitylated ID2 complex activates several endonucleases, such as FAN1, to stimulate unhooking processing of the ICL. (**d**) The unhooked ICL is removed by the NER pathway and bypassed by translesion synthesis polymerases REV1/pol ζ. (**e**,**f**) The HR pathway is recruited to repair the DSB on the other strand. After detection of DSB by PARP1, ADP-ribose molecules recruit the MRN complex to produce single-strand DNA and bind sister chromatid through a tail-to-tail link with another MRN complex. (**g**) Some FA members, such as BRCA1, BRCA2, BRIP1, and PALB2, in addition to the BCDX2 complex, induce the attachment of FANCR/RAD51 to ssDNA, which promotes strand invasion and DNA synthesis.

DNA crosslinking damages are caused by the covalent linkage between two nucleotides residing on either two complementary strands of DNA (inter-strand crosslink) or the same DNA strand (intra-strand crosslink) [34]. These crosslinks might arise from either exogenous sources such as mitomycin C and platinum-based chemotherapeutic agents or derive from endogenous metabolites comprising aldehydes and nitrous acid, to name a few [14,33]. Although intra-strand crosslinks are easily eliminated by the nucleotide excision repair (NER) pathway, inter-strand crosslinks must be repaired through the FA pathway involving several steps and proteins [14]. As shown in Figure 1, the proteins encoded by *FANC* genes in conjunction with several FA-associated factors collaborate to overcome inter-strand crosslink lesions [14]. The FA pathway triggers inter-strand crosslink repair principally during the S phase of the cell cycle by detecting two converging replication forks that formed the X shape construct near the inter-strand crosslink site [35]. In this procedure, FANCM, along with some other FA-associated proteins (FAAPs), senses the stalled replication fork on inter-strand crosslink damage. They act as the loading platform for assembling proteins of the FA core complex, including FANCA, B, C, E, F, G, L, M, T, and histone fold dimer proteins (MHF1, MHF2) (Figure 1a) [32,36]. The FA core complex as a ubiquitin-ligase conducts the formation and activation of FANCI-FANCD2 heterodimer (ID2 complex) through monoubiquitylation (Figure 1b) [37]. Monoubiquitylated ID2 in a process known as ‘unhooking’ governs nucleolytic incision at collapsed replication forks to cleave the inter-strand crosslink [14,36]. In this process, ubiquitylated FANCD2 recruits and activates several endonucleases comprising FANCP/SLX4, ERCC1-ERCC4 (ERCC4, also named FANCQ) heterodimers, and FAN1 (Fanconi-associated nuclease 1) to tackle the inter-strand crosslink lesion (Figure 1c) [32]. These endonucleases incise the ICL and leave it on one duplex while generating a double-strand break (DSB) on the other duplex [38]. Although the inter-strand crosslink is bypassed by translesion synthesis, the DSBs are repaired by HR (Figure 1d) [39]. For HR, a DSB is detected first by poly (ADP-ribose) polymerase 1 (PARP1) during the S phase. Then, PARP1 marks the lesion site by attaching ADP-ribose molecules to chromatin-bound proteins neighboring the break (Figure 1e) [40]. Consequently, ADP-ribose units recruit the MRE11-RAD50-NBS (MRN) complex to produce single-strand DNA (ssDNA) around the break (Figure 1f) [41]. Meanwhile, some FA components, including FANCS/BRCA1, FANCD1/BRCA2, BRIP1/FANCJ, and PALB2/FANCN, along with the RAD51B-FANCO/RAD51C-RAD51D-FANCU/XRCC2 (BCDX2) complex, promote the attachment of FANCR/RAD51 to the ssDNA overhangs. Finally, FANCR completes the HR repair via attacking the homologous DNA region (Figure 1g) [42]. Indeed, the FA pathway grants the high-fidelity repair of the ICL damages through blocking the error-prone NHEJ pathway and recruiting the FA-pathway-dependent HR repair [32,33].

Due to the importance of the HR pathway in cancer progression and drug resistance [43], the cancer relevance of *FANC* gene alterations at DNA and gene expression levels in various cancers has been considered. For example, germline mutations in *FANCD2*, the representative of the FA pathway, are likely linked with the increased risk of metastatic CRC [44]. The overexpression of *FANCD2* also predicts the increased probability of either lymph node metastasis or liver metastasis, which is reasonably associated with poor prognostic outcomes among CRC patients [45]. Since the monoubiquitylation of FANCD2 is a vital step in the FA pathway, its inhibition might selectively kill tumor cells [46]. All these observations highlight the conserved role of *FANC* genes in inter-strand crosslink repair.

### 2.2. FA Proteins Stabilize Stalled Replication Forks

One critical cellular process that contributes to DNA disruption is DNA replication. Upon DNA duplication, cells might encounter the challenges, such as stalled replication fork [47]. Replication fork slowing or stalling defines the replication stress, which causes chromosomal instability and tumor progression [48,49,50]. Recent data suggest that independent of inter-strand crosslink and HR repair, the FA proteins are essential to preserving genome stability upon replication stress [15]. Replication stress arises from endogenous or exogenous sources that interfere with the movement of the replication machinery and faithful duplication [48].

The principal endogenous source of fork stalling and DNA break is R-loop [21]. R-loops are three-stranded RNA:DNA structures mainly created from transcription-replication complex collision [51]. FA proteins actively protect cells from either R-loop formation or accumulation [52,53]. Using novel sequencing methods (DNA:RNA immunoprecipitation with deep sequencing), it was detected that *FANCD2* or *FANCA* deficiency causes a higher level of R-loop formation in murine or human cell lines [52]. Intriguingly, some investigations showed that FANCD2 overexpression limits replication stress and genome instability in *BRCA1/2*-deficient tumors [54]. Furthermore, FANCM helicase resolves R-loops obstacles via its translocase activity. It facilitates the displacement of RNA from R-loop structures in the absence of RNaseH (typical RNase to remove RNA-DNA hybrid) [55].

The G-quadruplex secondary structure (G4), guanine-rich regions of DNA that fold into four-stranded DNA structures, is another endogenous source of fork stalling that interferes with the progression of DNA replication [56]. The increased level of G-quadruplex structures has been diagnosed in various cancers that underlie genomic instability [57,58]. FANCJ helicase resolves the G4 DNA structure to support DNA replication and maintenance of genome integrity [59]. In such a scenario, FANCJ recognizes and unfolds the G4 structure through a specific motif, its helicase activity, in addition to recruiting REV1 polymerase [59,60]. Intriguingly, growing evidence has proposed that small molecules selectively bind and stabilize G4 DNA structures, which results in the inhibition of FANCJ unwinding activity on a variety of G4 DNA structures [61,62]. Therefore, DNA helicases might be potential targets in cancer therapy by exploiting the synthetic lethality approaches [63,64]. In this respect, Wu et al. [65] have shown that *FANCJ* deficiency leads to enhanced sensitivity to a G4-stabilizing ligand known as telomestatin (TMS). Similarly, a recent study reproduced these findings by using a G4-specific antibody. These findings demonstrated that exposure to TMS in FANCJ-deficient cells results in increased G4 formation and genomic instability [66].

Moreover, another source of fork stalling is dNTP pool depletion, which might be induced by chemotherapeutic agents such as hydroxyurea, an inhibitor of ribonucleotide reductase [67]. At high doses of hydroxyurea, *FANC* genes act to resolve the increased number of stalled replication forks. In such a situation, FANCD2 prevents MRE11-nascent DNA degradation. Moreover, FANCR/RAD51 and FANCD1/S (BRCA1/2) proteins promote the replication restart [68]. For instance, a cell lineage study showed the increased sensitivity to hydroxyurea in FANCR and FANCD2 knockdown and deficient cells [69].

All of the above studies verified that FA proteins are principally involved in DNA repair. Accordingly, unrepaired DNA lesions lead to genome instability, which fuels the initiation of malignancies. Considering the growing evidence suggesting the role of *FANC* genes in increased susceptibility to CRC, we provide an overview of *FANC* genes that have been proposed to predispose to CRC.

## 3. Potential Role of *FANC* Gene Mutations in Colorectal Cancer Susceptibility

**Figure 2:**
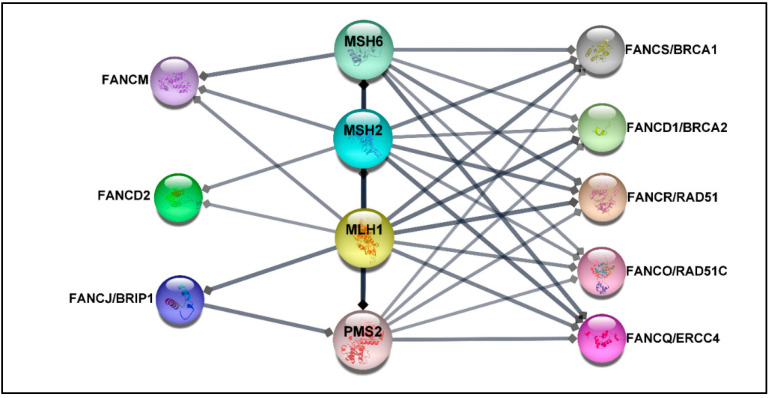
The protein–protein network. The protein–protein interaction (PPI) between MMR proteins comprising MLH1, MSH2, MSH6, PMS2, and 22 FA proteins was exported from the STRING database (high confidence (0.7)) [80] and visualized by using Cytoscape software, version 3.8.2 [81]. The association network shows that FANCS, FANCD1, FANCO, and FANCQ are linked with all selected MMR proteins, while FANCM interacts with MSH6, MSH2, and MLH1. FANCD2 is connected to MSH2 and MLH1, whereas FANCJ is associated with MLH1 and PMS2. The FA proteins without direct association with selected MMR proteins have been excluded.

Deficiency in MMR proteins has been generally used for CRC classification, while mutations in HR and *FANC* genes have been connected to hereditary breast-ovarian cancer syndrome [41] or have been reported in a higher percentage in metastatic castration-resistance prostate cancer compared with the localized disease [70]. Despite this fact, recent studies suggest that germline heterozygous mutations in some *FANC* genes increase the risk of developing CRC in non-FA patients [27,28,30,71,72,73,74]. Indeed loss-of-function mutations in HR genes result in the aggregation of DSB damages, hampering genomic stability and leading to cancer development. Although the proof of causation is not fully understood [5,41], it is well-established that MMR proteins, which play a crucial role in CRC, contribute to DNA double-strand HR repair [75,76]. In addition, previous findings have confirmed a direct interaction between MMR proteins and some FA proteins, such as FANCJ/MLH1 [77], FANCS/MSH2/MSH6/MLH2 [78], and FANCD2/MSH2/MLH1 [79] (see more details in Figure 2). Therefore, from this perspective, it is reasonable to speculate that PVs in *FANC* genes may be associated with the increased risk of CRC. Significantly, genetic testing of patients diagnosed with CRC not only can drive treatment but also facilitate earlier cancer screening for patients and their at-risk relatives. However, the optimal panel of genes for assessing the risk of CRC is not established yet.

### 3.1. Germline Monoallelic Mutations

As summarized in Table 1, among 22 *FANC* genes, *FANCS/BRCA1* and *FANCD2/BRCA2*, high-risk hereditary breast-ovarian cancer syndrome susceptibility genes, are the most investigated as CRC susceptibility genes beyond its well-known predispositions [27,28,29,30,31,44,73,82,83,84,85,86,87,88,89]. Recent findings revealed that the prevalence of *BRCA1/2* PVs among early-onset (1.3%) [30] and unselected patients with CRC (3.9%) [88] is more frequent than would be happening by chance. Likewise, the connection between the *FANCD1* mutation and increased risk of CRC has also been demonstrated among probands with familial CRC type X (2/48) [85]. For instance, in 2017, a prospective study of multigene panel testing by Pearlman et al. [30] examined the frequency of *BRCA1/2* mutations in 450 CRC patients younger than 50 years (early-onset CRC). The results indicated that six patients harbored mutations in *BRCA1/2* without changes in known CRC predispositions. Unexpectedly, two of them did not have a personal or familial history of breast or ovarian cancer. Likewise, Yurgelon et al. [27] assessed the prevalence of *BRCA1/2* mutations among 1058 unselected CRC patients. They also identified pathogenic mutations in *BRCA2* and *BRCA1* as the cause of CRC in eight and three individuals, respectively [27]. In addition, a large cohort study including 2398 unselected CRC patients demonstrated that the median age of CRC diagnosis in *BRCA1* mutation carriers was seven years lower than that of non-carriers, suggesting that *BRCA1* mutations might be linked with early-onset CRC [90]. Likewise, the notion of the association between *BRCA1* mutation and early-onset CRC has been verified in another international study involving a cohort of 7105 female patients harboring *BRCA1/2* PVs [91]. However, in contrast with these findings, a valuable investigation on a total of 6396 CRC tumor specimens did not find any significant association between *BRCA1/2* mutation and age [88]. Similarly, Cullinane et al. [92] presented a systematic review and meta-analysis including eleven studies for a total of 4831 CRC patients to assess the CRC risk in *BRCA* mutation carriers. Their fruitful study did not show any statistically significant increase in CRC development among *BRCA1/2* mutation carriers, regardless of the age or ethnicity of patients. Overall, despite all genuine attempts, the significance of BRCA mutation in CRC incidence remains controversial and, still, there are no specific guidelines or recommendations for gastric and bowel screening procedures for carriers of *BRCA1/2* mutations [93].

Besides *BRCA1/2*, the implication in CRC occurrence of *FANCJ/BRIP1* mutations, a moderate-risk factor for ovarian cancer, has attracted considerable attention [27,28,29,83,86,94,95]. As summarized in Table 1, recent studies detected that pathogenic germline *FANCJ/BRIP1* mutations are likely associated with the increased risk of developing CRC. For instance, Yurgelun et al. [27] and Gong et al. [29] have identified *FANCJ* PVs in 3 out of 1058 and 2 out of 618 unselected CRC patients, respectively. Likewise, the correlation between *FANCJ* mutation and increased risk of CRC has also been ascertained among familial CRC patients without germline PVs in known CRC predispositions (1/74) [28]. However, these reports are not sufficient to conclude on the role of *FANCJ* mutations in CRC, and further investigations are needed to ascertain if this association is significant.

Moreover, recent data have also suggested that pathogenic mutations in *FANCN/PALB2*, another established breast cancer predisposition gene, might also confer increased susceptibility to CRC [27,30,72,84]. For instance, a valuable study by AlDubayan et al. [72] evaluated the accumulation of germline *FANCN/PALB2* PVs among 680 CRC patients from two independent cohorts. To verify the findings of the study, they used the germline data of 1661 unselected CRC individuals, as well as 1456 early-onset CRC patients (age < 56 years). Their findings disclosed a significant enrichment (0.44%) of germline *FANCN/PALB2* PVs in three out of 680 unselected CRC patients versus the cancer-free control population. The enrichment was also verified in 1661 unselected CRC patients from the validation cohort (five individuals (0.3%)). On the other hand, the study failed to confirm a higher prevalence of *PALB2* PVs among 1456 early-onset CRC patients. This latter observation suggests that mutations in this gene predominantly cause late-onset CRC [72]. Nevertheless, Pearlman et al. [30] reported two positive individuals harboring PVs in *FANCN/PALB2* in a cohort of 450 early-onset CRC patients. Like *FANCJ/BRIP1*, PVs in *FANCN/PALB2* in CRC patients are moderately rare. Therefore, further investigations are needed to confirm the association of *FANCN/PALB2* mutations with the increased risk of CRC.

Likewise, next-generation sequencing studies manifest that harboring germline heterogenic mutations in other *FANC* genes may also confer increased susceptibility to CRC development. In this regard, exome sequencing studies have indicated that germline monoallelic mutations in *FANCC*, *FANCE* [28], *FANCM* [32,96], and *FANCA* [31] confer susceptibility to familial CRC (see Table 1). Alternatively, multigene panel testing studies highlighted that germline mutations in *FANCQ*, *FANCI*, *FANCL*, *FANCU*, *FANCO*, and *FANCD2* are likely linked with the increased incidence of metastatic CRC [44]. Nonetheless, it must be noted that, among the available studies, there are significant variations in the ratio of patients harboring even the same mutation. This occurrence might be due to the various methodological approaches used (e.g., PCR, arrays, different NGS platforms) and the consequent number of genes analyzed (e.g., candidate mutation screenings, panels including a variable number of genes, whole-exome sequencing) as well as to ethnic differences since several studies include mainly European populations [27,28,30,31,72,73,83,85,86,87,88,89,96], whereas some others considered Asian populations [29,44,84]. A summary of the associations between germline variations in *FANC* genes and CRC is presented in Table 1. However, further investigations are required to reach statistical significance at the population level and confirm the association of *FANC* genes with the increased risk of CRC development.

### 3.2. Somatic Mutations

**Figure 3:**
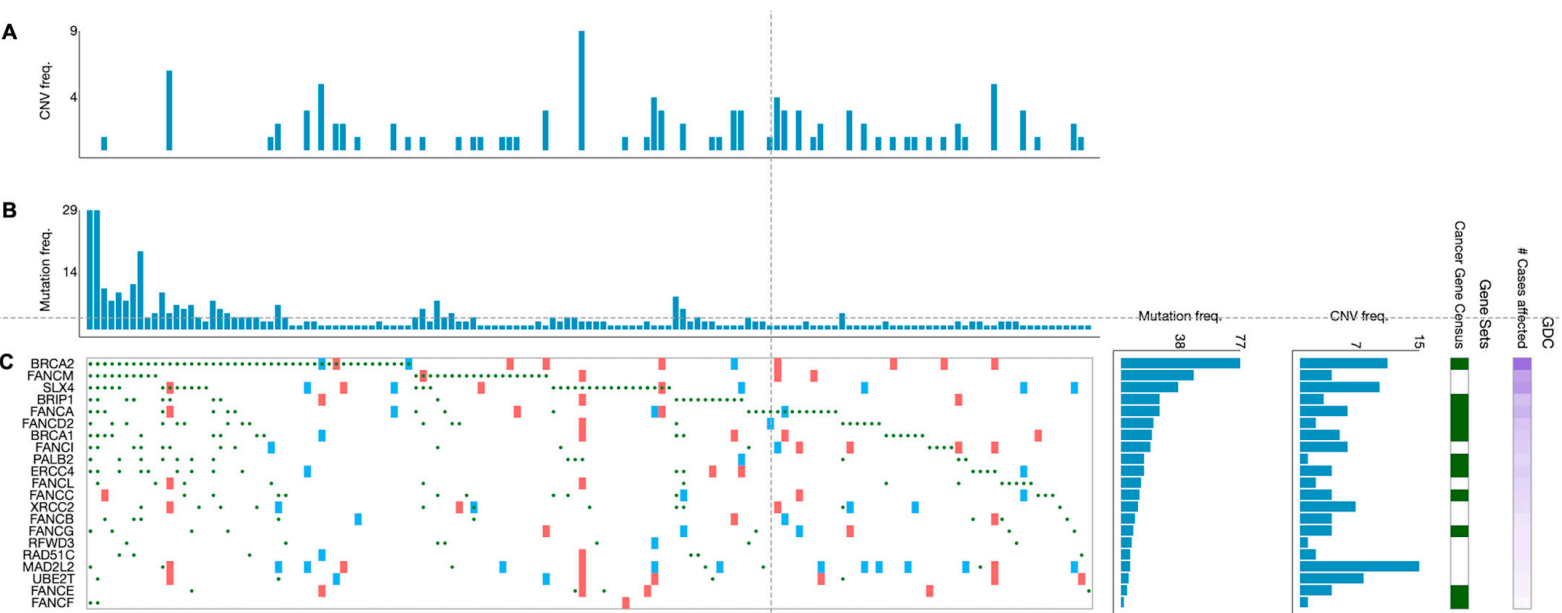
Genetic alterations of the FA genes in primary CRC specimens. Graphics show 139 primary CRC specimens displaying *FANC* gene mutations from a cohort of 395 cases. (**A**) Frequency of copy number variations and (**B**) frequency of mutations per tumor specimen. (**C**) Type of alterations per gene and per tumor specimen (green circles: missense, frameshift, start lost, stop lost, stop gained mutations; red squares: copy number variation (CNV) change, gain; blue squares: CNV change, loss). Data are from the TGCA program and have been generated from the GDC Data Portal [99].

Beyond germline mutations, somatically mutated *FANC* genes are frequently spotted in different cancerous tissue samples. An overview of cases in the NIH Genomic Data Commons (GDC) data portal has displayed that over 65% of tumors (without regards to tissue origin) harbor at least one mutation (any type) in one of the *FANC* genes [14]. However, the type of mutations (e.g., gain/loss of function mutations, deletions, amplifications) and their frequency vary widely among the *FANC* genes. Overall, according to the analysis of 395 primary CRC samples from TGCA projects, mutated *FANC* genes were detected in 139 tumor samples (35.2%) (Figure 3). In addition, the proportion of genetic alterations in *FANC* genes varied in such CRC samples in agreement with findings reported in other tumors [25]. For example, a wider analysis performed in a high number of patients (i.e., 3407 tumors of different origins) showed that most of the FA/HR components (e.g., *FANCS/BRCA1 FANCN/PALB2*, *FANCD1/BRCA2*) were more subjected to deletions and loss-of-function mutations, whereas the FA core complex (i.e., *FANCL* and *FANCT*/*UBE2T*) was predominantly affected by amplifications [25]. Intriguingly, the type of mutation probably has different functional and therapeutic consequences [25]. While somatic deletions and loss-of-function mutations in *FANC* genes are responsible for cancer transformation and progression, concurrently, they can provide sensitivity to DNA-damaging therapies [17,25]. A post-hoc analysis of metastatic CRC patients (*n* = 520) enrolled in the CALGB (Alliance)/SWOG 80,405 randomized phase III trial, performed by a next-generation sequencing approach, showed that patients treated with cytotoxic agents (i.e., FOLFOX or FOLFIRI) plus cetuximab, whose tumors harbored mutated *FANCD2*, had a worse overall survival compared with patients with wild-type *FANCD2* tumors [97]. Such observed worse outcome may be in keeping with the fact that, among the 22 *FANC* genes, *FANCD2* and *FANCD1/BRCA2* are classified as mutated cancer driver genes in CRC according to the IntoGen Compendium [98].

Overall, the knowledge of germline and somatic mutations has provided a new opportunity to develop appropriate screening guidelines and deliver proper treatment approaches. The information regarding *FANC* gene mutations might, in fact, result in a personalized plan for cancer prevention and early detection through offering novel diagnostic, prognostic, and predictive biomarkers [5,31,95].

## 4. FA Components as Potential Biomarkers for Predicting Disease Progression and Treatment Response

As discussed in Section 2.1, the FA pathway plays a crucial function in DNA DSB repair via HR. Alternatively, ionizing radiation [100] and DNA inter-strand crosslinking agents such as oxaliplatin and cisplatin prevent cell division and growth, principally by inducing DNA DSBs [14,101,102]. Based on these and other findings [103,104], it can be proposed that FA components in cancer cells play a critical role in the cellular capacity to repair DNA damages and chemo/radiotherapy response.

Recently, several studies have evaluated whether FA components can be served as potential biomarkers to either predict disease progression, govern treatment approaches, or offer novel targets for precision medicine [45,100,103,104,105,106,107,108]. In this regard, a recent study has evaluated the FANCT/UBE2T protein levels in 50 CRC biopsies compared to paired noncancerous mucosa from patients who did not receive radiotherapy or chemotherapy before surgery. The outcomes showed that the levels of FANCT protein in CRC specimens were higher than in paired noncancerous tissue. Likewise, tumor tissue samples with high FANCT protein expression were associated with advanced N staging, TNM staging, and worse overall survival compared to specimens with low UBE2T protein expression [106]. A further study confirmed these findings in 30 surgically resected CRC tumor samples compared with the adjacent colonic mucosa. Considering that only 10 out of 30 of these tumors were metastatic, it is conceivable that most of the tumor tissues analyzed did not receive chemotherapy before surgery. The study showed that higher *FANCT* tumor mRNA expression levels predicted a worse 5-year overall survival, and high FANCT tumor protein expression levels were associated with poor differentiation, as well as worse T and N classification [105]. In vitro results from this study also pointed out that the overexpression of *FANCT* enhanced p53 ubiquitination and degradation, while the knockdown of *FANCT* increased the apoptosis induction and reduced cell migration by decreasing the N-cadherin levels [105]. Considering the crucial function of FANCT/UBE2T in inter-strand crosslink repair through monoubiquitination and activation of the ID2 complex [109], these observations offer novel insights into UBE2T as a promising prognostic biomarker for CRC.

Moreover, several studies have reported that the FA components transform CRC progression from localized to migrative disease [45,104,106,107]. In this respect, the *FANCD2* mRNA expression levels have been evaluated in CRC samples from 133 patients collected at the surgery. Distant metastases were present in 18% of patients. The classification of tumor specimens according to the *FANCD2* expression levels indicated that tumor samples with high *FANCD2* mRNA levels were associated with worse 5-year overall survival, along with the increased likelihood of either lymph node metastasis or liver metastasis development [45]. Up-regulation of *FANCD2* at both mRNA and protein levels was also found in 56 CRC tumor samples compared with paired noncancerous colonic mucosa and was significantly correlated with the increased incidence of lymph node metastasis and a more advanced stage. Moreover, positive expression of FANCD2 protein was associated with worse 5-year overall survival [110]. These data suggest that *FANCD2* might be a valuable biomarker for CRC treatment management and its progression monitoring.

FANCU/XRCC2 mRNA and protein levels have been evaluated in a cohort of CRC patients whose bioptic tumor samples were collected prior to chemotherapy [107]. *FANCU/XRCC2* mRNA levels were significantly higher in CRC tissues compared with normal tissues. In the same study, in a second cohort of CRC patients, FANCU/XRCC2-positivity was found to be associated with a more advanced tumor stage and the increased incidence of either liver or lymph node metastasis [107]. Nevertheless, positive FANCU/XRCC2 protein expression predicted a smaller tumor size [107]. In addition, tumor FANCU/XRCC2-positivity correlated with a significantly poor response to 5-fluorouracil-based chemotherapy in terms of histological tumor regression grade [107]. This result found a counterpart in the in vitro phase of this study in which the knockdown of *XRCC2* in the SW480 human CRC cell line reversed 5-fluorouracil resistance by promoting the induction of apoptosis [107].

In a further study, FANCU/XRCC2 protein levels have been evaluated in tumor tissue from locally advanced rectal cancer (LARC) patients who did not undergo radiotherapy prior to surgery [100]. The study results showed that FANCU/XRCC2-positivity was significantly associated with advanced TNM staging. Moreover, FANCU/XRCC2 protein levels were directly correlated with radioresistance in LARC patients. Interestingly, also in this case, the authors showed that the knockdown of the *FANCU* sensitized SW480 human CRC cells to radiotherapy via impairing DNA DSB repair [100]. Since drug resistance can arise from HR renovation [43], *FANCU/XRCC2* as a crucial player in DNA DSB repair by HR might represent a valuable prognostic biomarker and predictive indicator of drug response in CRC [107] and LARC [100] patients.

Moreover, levels of *FANCR/RAD51* mRNA were analyzed among 48 CRC patients who underwent surgery without preoperative chemo/radiotherapy. The study results showed up to a 2.5-fold increase in *RAD51* mRNA levels in tumor samples compared to paired noncancerous tissue. The overexpression highlighted a correlation with advanced T staging [111]. Although this study failed to find relationships between overexpression of *RAD51* mRNA and disease progression, another study, by comparing the FANCR/RAD51 protein expression in bioptic tumor specimens from 1213 CRC patients, has revealed that high RAD51 protein levels predicted shorter overall survival compared with lower RAD51 expression (median overall survival 11 months versus 76 months, respectively) [108]. Ihara et al. [104] investigated the potential role of the expression levels of RAD51 protein in the prediction of response to oxaliplatin-based chemotherapy in unresectable CRC patients. Their results showed that high FANCR/RAD51 expression was correlated with worse progression-free survival [104]. Moreover, some in vitro studies have shown that an increase in RAD51 expression stimulated HR, leading to higher cellular resistance to the treatment with crosslinking agents or irradiation [112,113]. These results suggest that the expression level of FANCR/RAD51, as a crucial factor in HR repair, could represent a potential prognostic and predictive biomarker in CRC.

A further study investigated FANCJ/BRIP1 mRNA and protein levels in 219 CRC samples and paired noncancerous mucosa of patients who underwent surgery before receiving chemotherapy [103]. According to immunohistochemical staining, FANCJ/BRIP1 tumor expression levels were higher than those detected in normal mucosa. Moreover, high *FANCJ/BRIP1* tumor expression levels were associated with tumor depth, worse 5-year recurrence-free survival, and more importantly, resistance to 5-fluorouracil. In terms of tumor resistance, elevated *FANCJ/BRIP1* expression was connected to enhanced insensitivity, particularly in tumors with proficient MLH1 expression and not in MLH1-deficient tumors. The in vitro analysis in this study reproduced similar findings, underlining that high expression levels of *FANCJ/BRIP1* conferred 5-fluorouracil resistance to MLH1-proficient cells [103]. A possible explanation for this finding is that the physical interaction between FANCJ/BRIP1 and MLH1 is critical for FANCJ/BRIP1 localization to sites of either inter-strand crosslinks or DSB lesions. Indeed FANCJ–MLH1 interaction is required for cells to overcome the toxic effects of 5-fluorouracil. Thus, in the absence of MLH1, FANCJ/BRIP1 up-regulation cannot overtake inter-strand crosslinks [114]. These results are suggestive of the possibility that differential expression of *FANCJ/BRIP1* might serve as a predictive biomarker to drive personalized treatment strategies.

Overall, the above reported studies showed a poor prognosis in patients whose CRC expressed high mRNA or protein levels of *FANC* genes. These studies analyzed tumor specimens that, according to available information, did not receive preoperative chemotherapy. Thus, it is conceivable that high FANC transcript/protein levels may contribute to critical processes such as the increased DNA damage and genomic instability independently of the treatment with radiotherapy or chemotherapy.

Conversely, low BRCA1 or BRCA2 mRNA/protein expression levels have been substantially associated with poor prognosis in CRC [115,116,117,118]. A recent study that retrieved information from The Cancer Genome Atlas (TCGA) and other databases containing CRC patients has revealed an association between *BRCA1* mRNA-low tumor expression and worse clinicopathological features, including a higher proportion of advanced lymph node stages (N1/N2), a higher frequency of mucinous adenocarcinomas in conjunction with poor 5-year overall survival compared to the *BRCA1* mRNA-high expression group [116]. Similarly, one previous investigation has demonstrated that the down-regulation of *BRCA1* mRNA and protein expression in 120 CRC patients who underwent surgery without preoperative chemotherapy or radiotherapy correlated with advanced lymph node metastasis, TNM stage, shorter 5-year recurrence-free, and overall survival [117]. Wang et al. [119] evaluated BRCA1 protein expression with regards to subcellular localization. Their results showed that while low expression of cytoplasmic BRCA1 was associated with advanced TNM stage and worse overall survival, high expression of nuclear BRCA1 predicted poor outcomes in CRC patients. Beyond BRCA1, a few studies have considered the association between clinicopathological features and BRCA2 expression as an independent prognostic factor. Overall, these studies underlined that decreased BRCA2 expression in CRC was correlated with advanced TNM stage and poor differentiation, although no significant association between patient survival and BRCA2 has been reported [119,120]. These data suggested that BRCA1/2 might be a valuable prognostic biomarker in CRC besides its importance as a candidate to target therapy.

## 5. FA Components as Promising Therapeutic Targets in CRC

**Figure 4:**
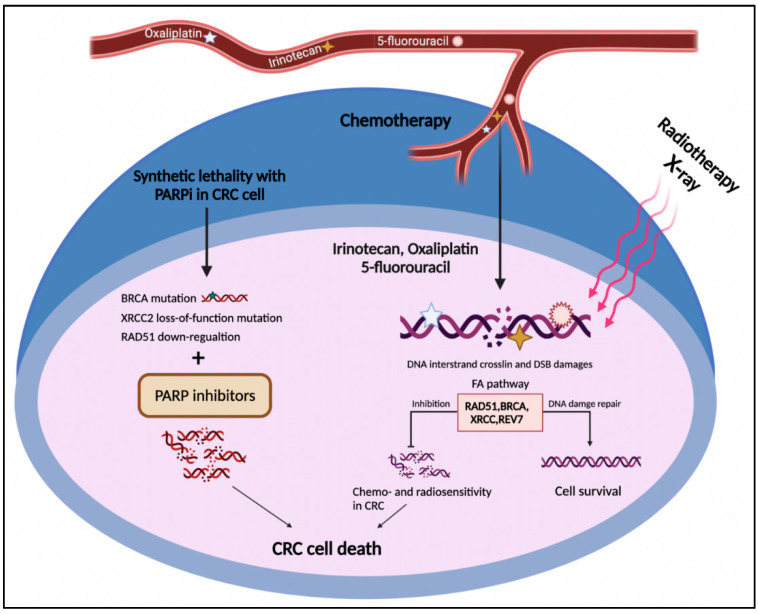
The FA pathway involvement in CRC treatment. The DNA damages caused by radio- and chemotherapy in CRC cells result in cell death. On the other side, up-regulation of the FA pathway components such as RAD51, BRCA1/2, XRCC2, and REV7 leads to HR restoration and drug resistance. The deficiency in the FA pathway components such as BRCA1/2, XRCC2, and RAD51 represents the synthetic lethal relationship with PARP1, which might introduce druggable candidates for PARP inhibitors in CRC cells.

The FA pathway is a potential target in cancer treatment through two distinct strategies. The first one is the chemosensitization of tumor cells to DNA crosslinking agents such as oxaliplatin and cisplatin. The second one exploits the synthetic lethal approach to selectively target tumor cells (Figure 4) [121,122]. The concept of synthetic lethality is defined as the inhibition of two or more genes leading to cell death, while repressing each individual gene does not lead to cell death. This approach is employed when the cancer cell has an initially deleterious mutation in a specific gene whose function is compensated by the second gene. Therefore, discovering and targeting the second gene may lead to cancer cell death [123].

According to the two above-mentioned strategies, the FA pathway as a substantial part of the DNA damage response system could serve as an approaching target for CRC intervention [46]. As mentioned above, CRC cells frequently develop resistance to DNA-damaging agents through HR renovation, including the FA pathway [43]. Indeed, increased activation of the FA repair system in CRC cells might neutralize the effect of first-line chemotherapeutic agents in CRC, comprising irinotecan, oxaliplatin, and 5-fluorouracil (Figure 4) [124]. As discussed in Section 2, FANC proteins not only cooperate in HR repair [125] but also contribute to replication stress tackling [126]. From a treatment perspective, although deficiencies in HR and collision in replication fork can be prominent causes of carcinogenesis, they are also promising targets for cancer treatment [127]. Emerging data have shown that targeting the components of the FA pathway not only sensitizes CRC cells to DNA-damaging agents but can also induce cell death through a synthetic lethal interaction. In this respect, *FANCR/RAD51* and *FANCD1/S* (*BRCA1/2*) genes received the most consideration in the context of the therapeutic vulnerability of CRC [128].

Recent discoveries pointed out that crosstalks between BRCA1, BRCA2, and RAD51 are essential to protect nascent DNA at stalled forks [67]. Upon replication stress, RAD51, regulated by the ataxia telangiectasia and Rad3-related kinase (ATR), preserves the stalled replication fork and promotes fork restart [47]. Accordingly, checkpoint kinase 1 (CHK1) directly interplays with ATR to assist replication fork stabilization during replication stress [41]. Hence, a collection of responses to replication stress is built on the ATR–CHK1 axis [129]. Increasing evidence shows that the ATR–CHK1 axis plays a crucial role in the viability of CRC cells by protecting replication forks under the condition of replication stress [130]. However, some CRC stem cells display resistance to ATR or CHK1 inhibitors. In such context, one study revealed that RAD51 targeting improves the vulnerability of CRC stem cells to the CHK1/2 inhibitor prexasertib and annihilates them through triggering mitotic catastrophe by the caspase-dependent mechanism [131]. Likewise, Manic et al. [129] have demonstrated that the RAD51 inhibitor B02, along with the MRE11 inhibitor mirin, selectively kills the PARP1-upregulated CRC stem cells via mitotic catastrophe without the need for exposing cells to ATR/CHK1 inhibitors [129]. In addition, a growing body of evidence shows that RAD51 up-regulation leads to PARP inhibitor (PARPI) resistance in CRC [132,133]. Regarding this, Smeby et al. [132] indicated that a subset of TP53 wild-type CRC cell lines were sensitive to the effect of PARPIs, whereas TP53 inhibition reversed the consequence. The authors of this study verified that talazoparib sensitivity in TP53 wild-type cell lines was correlated with down-regulation of FANCR/RAD51 protein expression. They highlighted a possible mechanism by which wild-type TP53 governs RAD51 suppression upon talazoparib treatment. Similarly, Arena et al. [133] showed that increased RAD51 foci formation, a functional biomarker of HR repair, was connected with resistance to the PARPI olaparib in patient-derived CRC models enriching for *KRAS* and *BRAF* mutations. In this study, the increase in RAD51 foci formation in all olaparib-resistant cells was confirmed, whereas a comparable effect was not observed in sensitive cells [133]. Regarding the association between *KRAS* mutations and RAD51 expression, Kalimutho et al. [134] revealed that ionizing radiations increased the generation of FANCR/RAD51 foci in the HCT116 *KRAS*-mutated CRC cells, compared with the wild-type counterparts. These authors showed that although *KRAS* mutation evokes replication stress and DNA damage accumulation, it assists cell surveillance by increasing the expression of RAD51. Consequently, they found that the co-inhibition of RAD51 and MEK1/2 signaling with the RAD51 inhibitor RI-1 and the MEK1/2 inhibitor AZD6244, respectively, induced DNA damage and apoptosis predominantly in *KRAS*-mutant cells while sparing wild-type cells. These findings support the hypothesis that the resistance of *KRAS*-mutant CRC cells to targeted agents can arise from the activation of compensatory pathways, particularly the HR pathway. Therefore, targeting RAD51 may offer a feasible strategy in killing KRAS-mutated CRC cells [134]. The findings of these recent works may support the candidature of the *FANCR/RAD51* gene as a predictor of sensitivity to PARPIs and HR functionality [132,133,134].

Aside from chemotherapeutic and targeted agents, it has been highlighted that some natural flavonoids, such as alpinumisoflavone, possess suppressing effects on RAD51. Li et al. [135] found that treatment with alpinumisoflavone, which shows antioxidant, anti-inflammatory, and anti-cancer properties, significantly decreased RAD51 levels in both in vitro and in vivo CRC models. Moreover, the knockdown of RAD51 increased the anti-cancer activity of alpinumisoflavone in preclinical CRC models, while the alpinumisoflavone effect was abolished by the up-regulation of RAD51 [135].

Knowledge of the *BRCA* status has raised hope for personalized cancer treatment by potentially adding platinum/DNA-damaging agents or PARPIs [129]. PARP is considered the potential synthetic lethal partner of *BRCA* mutations, and its inhibitors are now FDA-approved for the treatment of *BRCA1/2*-mutated breast and ovarian cancer [136]. In addition, the FDA also approved olaparib for the treatment of metastatic castration-resistant prostate cancer patients with deleterious HR gene mutations who progressed on a previous androgen receptor signaling inhibitor treatment [137]. Several studies propose that PARPIs prompt cell death by accumulating unrepaired DSBs due to the HR deficiency in *BRCA*-mutated cells. Even though *BRCA* mutations are infrequent in CRC, the PARPIs are undergoing several clinical trial investigations as single agents or combined with radiation or chemotherapeutic agents [138,139,140,141,142,143,144].

In this regard, Paviolo et al. [140] have shown that the treatment of BRCA-deficient CRC cell lines with olaparib may lead to genomic instability and cell death [140]. Moreover, Reisländer et al. [144] have revealed that the treatment of BRCA1- or BRCA2-deficient CRC cell lines with olaparib boosted the up-regulation of innate immune response genes upon intrinsically high levels of DNA damage [144]. Moving to the clinical setting, a phase II trial investigating the combination of the PARPI veliparib and the alkylating agent temozolomide showed good tolerability and clinical efficacy in patients with metastatic colon cancer [139]. Furthermore, the results of a phase 1b study have revealed that the administration of veliparib combined with neoadjuvant capecitabine and radiotherapy has an acceptable safety profile and a dose-proportional pharmacokinetic profile in patients with LARC [138]. A randomized phase II clinical trial showed similar efficacy for veliparib plus fluorouracil, leucovorin, irinotecan (FOLFIRI), and bevacizumab compared with FOLFIRI and bevacizumab in metastatic CRC patients [141].

Regarding the microsatellite status, Williams et al. [143] showed that the combination of irinotecan with niraparib, a potent PARP1/2I, in either MSI or MSS CRC cells, synergistically enhanced the anti-tumor effects of both agents either in vitro or in vivo [143]. Thus, these data suggest that niraparib enhances the effect of irinotecan regardless of microsatellite status [143].

Focusing on synthetic lethal interactions, Tiong et al. [145] evaluated in a publicly available CRC GEO dataset the gene expression of cancerous and normal tissues to screen novel synthetic lethal interactions in metastatic CRC. They introduced the *POLB* and *CSNK1E* genes, in addition to the *MSH2* gene, as potential synthetic lethal partners of *BRCA1* and *BRCA2*, respectively [145]. Such gene pairs (i.e., *BRCA1-POLB*, *BRCA1-CSNK1E*, and *BRCA2-MSH2*) were associated with clinicopathological features (i.e., tumor size, lymph node metastasis, and metastasis, respectively [145]. A further similar analysis performed on metastatic CRC cases suggested that BRCA1 targeting might increase the prognostic and therapeutic effects of bevacizumab [146]. In addition, polymerase theta (*POLQ*), which is involved in the repair pathway of replication-associated lesions, is introduced as the synthetic lethal partner of DNA damage repair genes such as *BRCA1/2* in various malignancies, including colon cancer. One possible mechanism to explain the synthetic lethality between *POLQ* and HR genes is that HR-deficient cancer cells rely on polymerase theta-mediated end joining (TMEJ) for their DNA DSB repair during replication stress [147].

As mentioned earlier, *BRCA* mutations can also help to personalize treatment by adding platinum/DNA-damaging agents. A case report described the addition of oxaliplatin to the chemoradiation regimen before surgery in a young patient with LARC who achieved a complete pathologic response without signs of disease recurrence at follow-up. The authors pointed out that the treatment modality was designed based on the confirmed *BRCA1* pathogenic mutation in the patient and known sensitivity of *BRCA*-mutant tumors to platinum-based chemotherapy [148].

FANCU/XRCC2 is not only a potentially useful predictive biomarker of treatment responses in CRC patients, as already detailed in Section 4 [100,107], but it might also be a promising therapeutic target for the treatment of CRC [145]. In this regard, Wang et al. [149] showed that the knockdown of *FANCU/XRCC2* expression in the T84 colon tumor cell line was associated with increased sensitivity to X-radiation in both in vitro and in vivo settings. The results represented *XRCC2* as a potential therapeutic target for overcoming radioresistance in CRC [149]. Another study showed that the up-regulation of *XRCC2* in CRC cell lines and tissue samples was correlated with the down-regulation of miR-7 [147]. Moreover, *XRCC2* targeting by miR-7 could efficiently induce apoptosis and inhibit proliferation in several CRC cell lines [150]. Additionally, according to the synthetic lethality concept, tumors showing *XRCC2* loss-of-function mutations could be responsive to PARP inhibition [151]. In support of this hypothesis, cells harboring the biallelic mutation of *FANCU/XRCC2* showed increased sensitivity to olaparib compared to *XRCC2* wild-type cells [152]. Thus, the identification of breast cancer patients and perhaps other cancer patients with deleterious somatic variants of *XRCC2* may be a basis for such personalized treatments. On the contrary, in a study by Xu et al. [153], CRC cell lines with a higher expression of XRCC2 were sensitive to olaparib, in contrast with the theory of synthetic lethality. The authors claimed that one possible explanation for their finding was the involvement of both XRCC2 and PARP1 in the HRR pathway in which XRCC2 expression is required for the effect of olaparib [153].

FANCV/REV7, a key component of translesion synthesis polymerase zeta, can potentially be targeted to overcome CRC chemoresistance. A study by Sun et al. [154] assumed that the down-regulation of FANCV/REV7 reverted 5-fluorouracil and oxaliplatin resistance in CRC cells by impairing the translesion DNA synthesis pathway. In vitro data showed that the knockdown of *FANCV/REV7* sensitized CRC cells to 5-fluorouracil and oxaliplatin, which was also confirmed in a murine xenograft model [154]. These observations suggest that FANCV/REV7 can be a promising target to overcome drug resistance by producing synergistic effects with first-line chemotherapeutic agents in CRC.

Given that drug resistance is a critical obstacle in CRC treatment, the knowledge of such mutations could allow for the proper adding of platinum/DNA-damaging agents or PARPIs in the neoadjuvant setting for each patient. However, more comprehensive investigations are required for the *FANC* genes targeting in the clinic. A synthesis of the above-reported studies is provided in Table 2, in which the FA components, their status, and drug treatment or synthetic lethality partners are described.

## 6. Conclusions

The best intention of this review was to highlight the importance of *FANC* genes as potent predisposing risk factors to CRC and their potential applications in future clinical practice. Available data suggest that *FANC* genes could be used in CRC management as potential biomarkers for early diagnosis, evaluating prognostic outcomes, monitoring therapy response, modifying treatment plans, and selecting effective therapeutic agents.

## Figures and Tables

**Table 1:** Germline mutations in *FANC* genes among CRC patients without mutations in known CRC predisposition genes.

Gene(s) and No.	Study Group	Study Method	N. of *FANC* Gene Mutation Carriers in CRC Patients	Reference
*FANCD1/BRCA2*: 2	2 patients from 1 family, no mutations in known CRC genes	Point mutation screening of the *BRCA1* and *BRCA2* genes	2/2	[73]
*FANCD1/BRCA2*: 2	48 FCCTX probands with strong familial CRC aggregation; no mutations in known CRC genes	Mutation screening of *BRCA2*	2/48	[85]
*FANCD1/BRCA2*: 9 *FANCS/BRCA1*: 6 *FANCJ/BRIP1*: 2	1260 CRC patients with suspected Lynch syndrome	25-gene NGS panel testing	17/1260	[86]
*FANCS/BRCA1*: 4 *FANCD1/BRCA2*: 1 *FANCN/PALB2*: 2	450 EOCRC patients	25-gene NGS panel testing	7/450	[30]
*FANCS/BRCA1*: 1	430 EOCRC patients < 50 years	154-gene NGS panel testing	1/430	[87]
*FANCI*:1, *FANCL*: 1 *FANCO/RAD51C*: 1 *FANCQ/ERCC4*: 1 *FANCS/BRCA1*: 1 *FANCU/XRCC2*: 1	330 mCRC patients age ≤ 55 years, 110 mCRC patients age > 55 years	98-gene NGS panel testing	6/440	[44]
*FANCD1/BRCA2*: 179 *FANCS/BRCA1*: 72	6396 unselected CRC samples	592-gene NGS panel testing	251/6396	[88]
*FANCD1/BRCA2*: 8 *FANCJ/BRIP1*: 3 *FANCS/BRCA1*: 3 *FANCN/PALB2*: 2	1058 unselected CRC samples	25-gene NGS panel testing	16/1058	[27]
*FANCN/PALB2:* 3	680 unselected CRC patients	40-gene NGS panel testing	3/680	[72]
*FANCD1/BRCA2*: 1 *FANCS/BRCA1*: 1	618 unselected CRC patients	73-gene NGS panel testing	2/618	[29]
*FANCJ/BRIP1*: 3 *FANCD1/BRCA2*: 2 *FANCS/BRCA1*: 2 FANCU/XRCC2: 1	189 unselected CRC patients	25-gene NGS panel testing	8/189	[83]
*FANCD1/BRCA2*: 1 *FANCN/PALB2*: 1	88 EOCRC patients ≤ 50, MMR-proficient	WES	2/88	[84]
*FANCD1/BRCA2*: 4 *FANCJ/BRIP1*: 1 *FANCO/RAD51C*: 1	133 EOCRC patients < 55 years	WES	6/133	[89]
*FANCD1/BRCA2*: 2 *FANCC*: 1, *FANCE*: 1 *FANCJ/BRIP1*: 1	74 CRC patients from 40 unrelated families with strong CRC aggregation; no mutations in known CRC genes	WES	5/74	[28]
*FANCM:* 4	94 CRC patients (47 CRC-affected cousin pairs)	WES	4/94	[96]
*FANCA*: 1 *FANCD1/BRCA2*: 1 *FANCD2*: 1, *FANCM*: 2	141 unselected CRC patients	WES	5/141	[31]

N: number of patients; CRC: colorectal cancer; EOCRC: early-onset CRC (patients age ≤ 50); mCRC: metastatic CRC; FCCTX: familial CRC type X (Lynch syndrome without mutations in MMR genes); MMR: mismatch repair; NGS: next-generation sequencing; WES: whole-exome sequencing.

**Table 2:** FA components as potential therapeutic targets in CRC.

FA Component	FA Component Status	Drug Treatment(s) or Synthetic Lethality Partner(s)	Study Materials	Setting	Mechanism of Action/Observed Results	Ref.
*FANCR/RAD51*	RAD51 inhibition (B02 inhibitor)	Prexasertib (CHK1/2 inhibitors)	CRC stem cells	In vitro	Triggering mitotic catastrophe	[131]
	RAD51 inhibition (B02 inhibitor)	Mirin (MRE11 inhibitor)	PARP1-upregulated CRC stem cells	In vitro and in vivo	Triggering mitotic catastrophe	[129]
	Decreased RAD51 protein	Talazoparib (PARP inhibitor)	*TP53* wild-type cell lines	In vitro	Increased sensitivity to the PARP inhibitor	[132]
	Increased RAD51 foci formation	Olaparib (PARP inhibitor)	Patient-derived CRC models	In vitro	Resistance to the PARP inhibitor	[133]
	RAD51 inhibition (RI-1 inhibitor)	AZD6244 (MEK1/2 inhibitor)	*KRAS*-mutant cells	In vitro	Induction of DNA damage and apoptosis	[134]
	*RAD51* knockdown (specific siRNA)	Alpinumisoflavone (natural flavonoid)	CRC cell lines	In vitro	Increased anti-cancer activity of alpinumisoflavone	[135]
*FANCD1/S (BRCA1/2)*	*BRCA1/2* depletion (specific shRNA)	Olaparib (PARP inhibitor)	BRCA-deficient cell lines	In vitro	Genomic instability and cell death	[140]
	*BRCA1/2* depletion (specific shRNA)	Olaparib and talazoparib (PARP inhibitors)	BRCA-deficient cell lines	In vitro and in vivo	Elicit innate immune response	[144]
	ND	Niraparib (PARP inhibitor) and irinotecan	MSI or MSS CRC cells	In vitro or in vivo	Enhancement of the anti-tumor effects of both agents	[143]
	*BRCA1* gene expression	*POLB, CSNK1E, MSH2*	GEO datasets-mCRC patients and CRC cells	Translational and in vitro	Synthetic lethality	[145]
	*BRCA2* gene expression	*MSH2*	GEO datasets-mCRC patients and CRC cells	Translational and in vitro	Synthetic lethality	[145]
	High *BRCA1* gene expression	Bevacizumab (VEGF Inhibitor)	GEO datasets-mCRC patients	Translational	More favourable PFS	[146]
	Mutated *BRCA1*	Oxaliplatin plus radiation before surgery	One LARC patient	Case report	Increased sensitivity to platinum-based chemotherapy	[148]
*FANCU/XRCC2*	*XRCC2* depletion (specific shRNA)	X-radiation	T84 colon tumor cell line	In vitro and In vivo	Increased sensitivity to X-radiation	[149]
	*XRCC2* targeting by miR-7	-	CRC cell lines	In vitro	Apoptosis and inhibition of proliferation	[151]
	Biallelic mutated *XRCC2*	Olaparib (PARP inhibitor)	Fibroblast cells	In vitro	Increased sensitivity to olaparib	[152]
	Increased expression of XRCC2	Olaparib (PARP inhibitor)	CRC cell lines	In vitro	Synthetic lethality	[153]
*FANCV/REV7*	REV7 depletion (CRISPR/Cas9)	5-fluorouracil and oxaliplatin	CRC cells	In vitro and In vivo	Impair of translesion DNA synthesis pathway	[154]

ND, not determined; CRC, colorectal cancer; LARC, locally advancer rectal cancer; GEO, Gene Expression Omnibus; PFS, progression-free survival

## Data Availability

Not applicable.

## Abbreviations

APC	APC regulator of WNT signaling pathway
ATR	ataxia telangiectasia and RAd3-related kinase
BCDX2 complex	RAD51 paralogs (RAD51B, RAD51C, RAD51D) and XRCC2
BMPR1A	bone morphogenetic protein receptor type 1A
BRAF	B-Raf proto-oncogene, serine/threonine kinase
BRCA	BRCA1 DNA repair associated (alias FANCS)
BRCA2	BRCA2 DNA repair associated (alias FANCD, FANCD1)
BRIP1	BRCA1 interacting helicase 1 (alias FANCJ)
CHK1	checkpoint kinase1
CNV	copy number variation
CRC	colorectal cancer
dNTP	deoxyribonucleoside triphosphate
DSB	double-strand break
EOCRC	early-onset CRC
ERCC1	ERCC excision repair 1, endonuclease catalytic subunit
ERCC4	ERCC excision repair 4, endonuclease catalytic subunit (alias FANCQ)
FA	Fanconi anemia
FAAP	RNA 2′,3′-cyclic phosphate and 5′-OH ligase
FAAP100	FA core complex associated protein 100
FAAP20	FA core complex associated protein 20
FAAP24	FA core complex associated protein 24
FAN1	FANCD2 and FANCI associated nuclease 1
FANC	FA complementation group
FANCA	FA complementation group A
FANCB	FA complementation group B
FANCC	FA complementation group C
FANCD1	BRCA2 DNA repair associated (official symbol BRCA2)
FANCD2	FA complementation group D2
FANCE	FA complementation group E
FANCF	FA complementation group F
FANCG	FA complementation group G (alias XRCC9)
FANCI	FA complementation group I
FANCJ	BRCA1 interacting helicase 1 (official symbol BRIP1)
FANCL	FA complementation group L
FANCM	FA complementation group M
FANCN	partner and localizer of BRCA2 (official symbol PALB2)
FANCO	RAD51 paralog C (official symbol RAD51C)
FANCP	SLX4 structure-specific endonuclease subunit
FANCQ	ERCC excision repair 4, endonuclease catalytic subunit (official symbol ERCC4)
FANCR	RAD51 recombinase (official symbol RAD51)
FANCS	BRCA1 DNA repair associated (official symbol BRCA)
FANCT	FA complementation group T/Ubiquitin Conjugating Enzyme E2 T (official symbol UBE2T)
FANCU	X-ray repair cross complementing 2 (official symbol XRCC2)
FANCV	mitotic arrest deficient 2 like 2 (official symbol: MAD2L2; alias REV7)
FANCW	ring finger and WD repeat domain 3 (official symbol RFWD3)
FCCTX	familial CRC type X
FOLFIRI	fluorouracil/leucovorin, irinotecan
FOLFOX	fluorouracil/leucovorin, oxaliplatin
G4	G-quadruplex
GEO	Gene Expression Omnibus
HR	homologous recombination
HRR	homologous recombinational repair
ICL	inter-strand crosslink
ID2 complex	FANCI-FANCD2
KRAS	Kirsten RAS proto-oncogene
LARC	locally advanced rectal cancer
mCRC	metastatic CRC
MEK1	mitogen-activated protein kinase kinase 1 (official symbol MAP2K1)
MEK2	mitogen-activated protein kinase kinase 2 (official symbol MAP2K2)
MHF1	centromere protein S (official symbol CENPS)
MHF2	centromere protein X (official symbol CENPX)
MLH1	mutL homolog 1
MLH2	PMS1 homolog 1, mismatch repair system component (official symbol PMS1)
MMR	mismatch repair,
MRN complex	MRE11-RAD50-NBS proteins
MSH2	mutS homolog 2
MSH6	mutS homolog 6
MSI	microsatellite instability
MSS	microsatellite stability
MUTYH	mutY DNA glycosylase
NCI GDC	National Cancer Institute-Genomic Data Commons
NER	nucleotide excision repair
NGS	next-generation sequencing;
NHEJ	non-homologous end joining
NTHL	nth like DNA glycosylase
PALB2	partner and localizer of BRCA2 (alias FANCN)
PARP1	poly (ADP-ribose) polymerase 1
PARPi	PARP inhibitor
PFS	progression-free survival
PMS2	PMS1 homolog 2, mismatch repair system component
POLD1	DNA polymerase delta 1, catalytic subunit
POLE	DNA polymerase epsilon, catalytic subunit
POLQ	DNA polymerase theta
PPAP	polymerase proofreading–associated polyposis
PPI	protein–protein interaction
PTEN	phosphatase and tensin homolog
PV	pathogenic variation
RAD51	RAD51 recombinase (alias FANCR)
RAD51B	RAD51 paralog B
RAD51C	RAD51 paralog C (alias FANCO)
RAD51D	RAD51 paralog D
REV1	REV1 DNA directed polymerase
REV7	mitotic arrest deficient 2 like 2 (official symbol: MAD2L2; alias FANCV)
RFWD3	ring finger and WD repeat domain 3 (alias FANCW)
RNaseH	ribonuclease H
SMAD4	SMAD family member 4
ssDNA	single-strand DNA
STK11	serine/threonine kinase 11
TLS	translesion DNA synthesis
TMEJ	polymerase theta-mediated end joining
TMS	telomestatin
TNM	tumor (lymph) node metastasis staging
TP53	tumor protein p53
UBE2T	Ubiquitin Conjugating Enzyme E2 T (alias FANCT)
VEGF	Vascular endothelial growth factor
WES	whole-exome sequencing
XRCC2	X-ray repair cross complementing 2 (alias FANCU)
